# Early application of CVVH In the complex treatment of patients with early severe acute pancreatitis

**DOI:** 10.1186/cc10969

**Published:** 2012-03-20

**Authors:** I Aleksandrova, M Ilynsky, S Rei, G Berdnikov, L Marchenkova, V Kiselev

**Affiliations:** 1 Hospital Research Institute for Emergency Medicine N.V. Sklifosovsky, Moscow, Russia

## Introduction

A large population-based study of 1,024 deaths from acute pancreatitis (AP) has revealed that the median time lapse between the onset of AP and death was 6 days [1]. A number of authors considered the patients with persistent or progressive early multiple organ failure (MOF) as patients with early severe acute pancreatitis (ESAP) [2].

## Methods

The aim of current study was to evaluate the efficiency of early CVVH in a complex treatment of ESAP. The retrospective analysis involved 106 patients. The patients were divided into three groups: the first group (*n *= 45) received CVVH dose <30 ml/kg/hour, the second group (*n *= 20) received the dose >30 ml/kg/hour, and in the third group (*n *= 41) CVVH was not used during the early phase of disease (Table 1). In the first and second groups the median time interval between admission and start of CVVH was 2 (2; 3) days.

**Table 1:** 

Variable	First group	Second group	Third group
Age	42 ± 15	39 ± 13	47 ± 16
BMI	31 ± 5	29 ± 4	29 ± 5
APACHE II score	17 (5)	17 (9)	15 (7)
SOFA score	5 (4)	5 (3)	5 (3)
Ranson score	8 (6)	8 (7)	10 (9)
Early mortality (%)	27	10*	42
Infection (%)	47	35	29
Overall mortality (%)	49	25*	51

Data presented as median (IQR). *P < 0.05, second group versus third group.

## Results

As compared to reference group 3, significant (*P *= 0.022) reduction of early mortality (14 days) was observed in the second group, and decreasing tendency (*P *= 0.093) of mortality rate was detected in the first group. The median time interval between admission and death was 14 days (in the first and second groups) and 5 days in the third group.

## Conclusion

The early application of the CVVH increases time interval for care delivery and allows reducing early mortality. The best results were obtained in the group of patients who were treated with the higher dose of CVVH (earlier restoration of homeostasis and decreased severity of early MOF).
